# Targeted Nanoparticles for Fluorescence Imaging of Folate Receptor Positive Tumors

**DOI:** 10.3390/biom10121651

**Published:** 2020-12-09

**Authors:** Aimee J. Marko, Ballav M. Borah, Kevin E. Siters, Joseph R. Missert, Anurag Gupta, Paula Pera, Meden F. Isaac-Lam, Ravindra K. Pandey

**Affiliations:** 1 Photodynamic Therapy Center, Cell Stress Biology, Roswell Park Comprehensive Cancer Center, Buffalo, NY 14263, USA; aimee-marko@mail.com (A.J.M.); joseph.missert@roswellpark.org (J.R.M.); anurag.gupta@roswellpark.org (A.G.); paula.pera@roswellpark.org (P.P.); 2 Photolitec, LLC, Buffalo, NY 14203, USA; ballav.borah@roswellpark.org (B.M.B.); kevin.siters@Photolitec.org (K.E.S.); 3 Department of Chemistry and Physics, Purdue University Northwest, Westville, IN 46391, USA; isaaclam@pnw.edu

**Keywords:** polyacrylamide-based nanoparticles, drug delivery, folate receptor target-specificity

## Abstract

This report presents the synthesis and folate receptor target-specificity of amino-functionalized polyacrylamide nanoparticles (AFPAA NPs) for near-infrared (NIR) fluorescence imaging of cancer. For the synthesis of desired nano-constructs, the AFPAA NPs (hereafter referred to as NPs) were reacted with a NIR cyanine dye (CD) bearing carboxylic acid functionality by following our previously reported approach, and the resulting conjugate (NP-CD) on further reaction with folic acid (FA) resulted in a new nano-construct, FA-NP-CD, which demonstrated significantly higher uptake in folate receptor-positive breast cancer cells (KB+) and in folate receptor over-expressed tumors in vivo. The target-specificity of these nanoparticles was further confirmed by inhibition assay in folate receptor-positive (KB+) and -negative (HT-1080) cell lines. To show the advantages of polyacrylamide (PAA)-based NPs in folate receptor target-specificity, the CD used in preparing the FA-NP-CD construct was also reacted with folic acid alone and the synthetic conjugate (CD-FA) was also investigated for its target-specificity. Interestingly, in contrast to NPs (FA-NP-CD), the CD-FA conjugate did not show any significant in vitro or in vivo specificity toward folate receptors, showing the advantages of PAA-based nanotechnology in delivering the desired agent to tumor cells.

## 1. Introduction

Medical imaging has become extremely important in the early detection, diagnosis, and treatment of cancer [1]. After cancer has been diagnosed, imaging is often used to follow the course of treatment to monitor growth or remission [2]. In recent years, in vivo fluorescence imaging has been used in determining the localization of cancer and in image-guided surgery [3]. A large number of near-infrared (NIR) dyes, especially cyanine dye (CD), have been evaluated for imaging tumors in mouse models [4,5,6,7,8]. Among the well-established techniques, e.g., computed tomography (CT), magnetic resonance imaging (MRI), positron emission tomography (PET), X-ray, and ultrasound (US), are very important tools that can be used in the fight against deeply seated cancer. However, these techniques lack real time capabilities, which could be extremely useful during surgery in differentiating malignant from normal tissue. Sevick-Muraca et al. [9] and others [10] have shown the utility of indocyanine dye, a type of CD, for the diagnostic imaging of breast cancer and fluorescence-enhanced optical tomography via phantom studies.

Significant advances have been made in designing instruments for in vivo fluorescence imaging in animal models [11]. For example, epi- and transillumination fluorescence imaging offers a practical and sensitive platform for in vivo imaging. However, its application is limited to qualitative observations due to its inability to resolve depth or account for the nonlinear dependence of light on depth and on tissue optical properties. Another imaging method that has been more recently developed is fluorescence molecular tomography (FMT) [12], which can resolve and quantify fluorochromes deep in tissues through the use of tomographic principles. These approaches employ mathematical models that describe light propagation in tissue for yielding the three-dimensional (3D) tomography of tissues based on diffused light. Similarly, in transillumination, most of the attention has been focused on using light in the near-infrared (NIR) region due to the greater depth of penetration. However, whole-body fluorescence imaging is still in the pre-clinical stage. This technique alone or in combination with clinically available instruments could be extremely useful in imaging and monitoring the tumor response of various cancer types, e.g., brain, head and neck, prostate, and breast cancers.

Porphyrins or reduced porphyrins (chlorins and bacteriochlorins) are highly fluorescent molecules, and this inherent property has been explored in the fluorescence-guided photodynamic therapy (PDT) of superficial cancers [13]. Among the photosensitizers (PSs) derived from naturally occurring chlorophyll-a and bacteriochlorophyll-a, certain alkyl/aryl ether derivatives of pyropheophorbide-a [14,15,16,17,18] and bacteriopurpurinimides [19] are of special interest due their desired photophysical properties, high tumor uptake, and limited toxicity, including skin phototoxicity [20]. Most tetrapyrrole-based compounds are water-insoluble [21,22,23]; therefore, a variety of polymers and nanoparticles based on these compounds are being used as drug delivery vehicles [24,25,26,27].

Among the nanoparticles (NPs) investigated in our laboratory [28,29,30], non-toxic polyacrylamide (PAA) NPs are of special interest due to their biocompatibility and biodegradability [31,32,33,34]. Therefore, it was decided to explore their utility in developing multifunctional platforms for cancer imaging with an option of therapy. To explore the utility of PAA NPs as bifunctional agents, a post-loading method was applied to incorporate 3-(1’-hexyloxy)ethyl-3-devinyl pyropheophorbide-a HPPH and CD [35]. Pre-formed 30–35 nm PAA NPs were sequentially incubated with HPPH and CD. Non-associated HPPH and CD were removed by 100 kDa size exclusion filtration. The precise nature of the association of HPPH and CD with PAA NPs remains to be determined. A 2:1 HPPH/CD ratio produced optimal tumor imaging and long-term tumor cure. In contrast to the HPPH–CD conjugate, no quenching was observed in this nano-formulation. The data clearly illustrated the advantage of post-loading, which reduces the therapeutic dose compared to that of the HPPH–CD conjugate formulated in 1% Tween-80/water [36]. To investigate the toxicity of PAA NPs, these were injected into BALB/c mice with an up to 80-fold higher dose than the dose required for post-loading the imaging (CD) and PDT agents (HPPH). No weight loss or organ toxicity was observed over the 30-day post-injection period.

The strategies presented in this article were aimed to develop an efficient synthetic strategy for incorporating folic acid moieties at the periphery of an NP–CD conjugate and to investigate its folate receptor target specificity and imaging of folate receptor-positive tumors subcutaneously implanted in mice.

## 2. Methods

### 2.1. Preparation of Polyacrylamide Nanoparticles (PAA NPs)

**Scheme 1:**
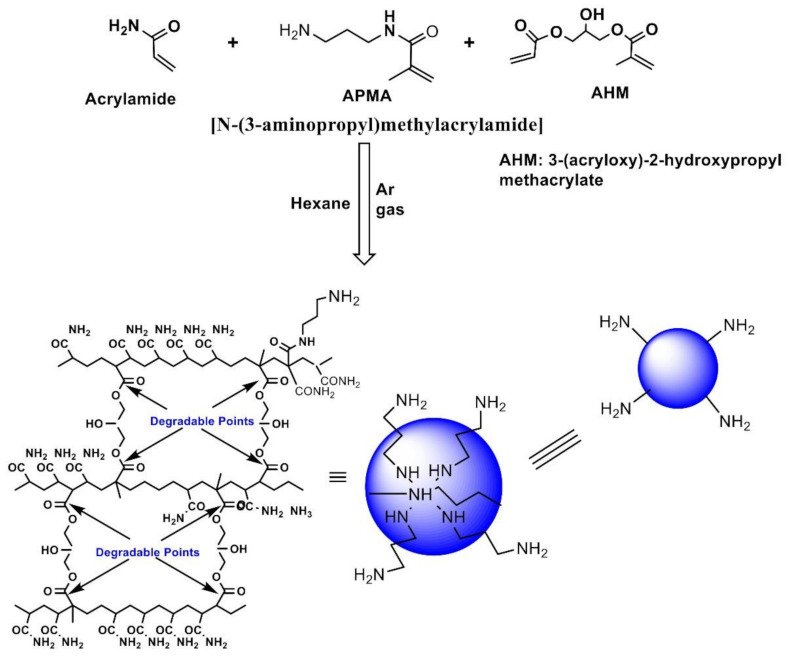
Preparation of amino-functionalized polyacrylamide nanoparticles (Gupta et al., 2012 [34]).

For the preparation of amino-functionalized NPs [34] (Scheme 1), anhydrous hexane (45 mL) was stirred under Ar for 1 h at room temperature. Dioctyl sulfosuccinate sodium salt (1.6 g; 3.6 mmol) was added and the Ar atmosphere was restored. Then, 3.3 mL of Brij-30 was added with a syringe to the reaction flask and the mixture was stirred for 20 min under a constant purge of argon. To this mixture, a solution of acrylamide (711 mg; 10.0 mmol) and N-(3-aminopropyl)methacryamide HCL (89 mg; 0.50 mmol) in 1.3 mL of 18 MΩ argon-purged water and 3-acryloxy-2-hydroxypropyl methacrylate (375 µL; 428 mg; 2.0 mmol) were added. The resulting solution was transferred into a reaction flask with a syringe was mixed for 20 min under a constant purge of argon.

Polymerization was initiated by adding freshly prepared ammonium persulfate (100 µL of a 10% *w*/*v* aqueous solution in 18 MΩ argon-purged water). After 30 min, N,N,N′,N′-tetramethylethylene diamine (100 µL) was added. The solution was stirred at room temperature for 2 h. The reaction was exposed to an ambient atmosphere to stop the polymerization reaction. The turbid hexane solution was evaporated by rotary evaporation and a white solid was obtained. Next, 95% ethanol (50 mL) was added to the flask and sonicated for 2 min. The slurry was transferred to an Amicon ultra-filtration cell equipped with a Biomax 500 kDa cut-off membrane and EtOH was added up to 100 mL. Then, 5–10 psi pressure was applied using Ar and the slurry was stirred. The liquid run off was collected below the cell and disposed of. The slurry was washed a total of 5 times (5 × 100 mL) with ethanol and then washed with 18 MΩ water (5 × 100 mL). The concentrated nanoparticles were lyophilized overnight and stored at −20 °C.

### 2.2. Preparation of NPs Conjugated with a Cyanine Dye (PAA NP–CD)

The methodology used previously for the preparation of title NP [36] was slightly modified. In brief, 50 mg of lyophilized PAA nanoparticles were suspended in 3 mL of phosphate buffer solution (PBS × pH 7.4) and sonicated for 10 min, resulting in a milky suspension. Then, 11 mg (57 μmol) of EDCI and 32 mg of Sulfo-NHS (147 μmol) were dissolved in 1.9 mL of MES Buffer (100 mM, pH 6.0). To this solution, 4 mg (4.1 μmol) of CD, dissolved in 100 μL of DMSO, was added and stirred at room temperature for 20 min. Then, 100 μL of 1 N NaOH was added to this PAA nanoparticle solution to bring the final pH of the solution to 7 and was stirred for 2 h at room temperature, protected from light. The resulting mixture was transferred to an Amicon Ultra-4 100 kDa centrifuge filter device and was centrifuged for 40 min at 4000 RPM to remove any DMSO, isourea by-product, or unreacted cyanine dye. The remaining pellet was dispersed in 500 μL of 18 MΩ water using repeated pipetting and a spatula, diluted with 200-proof ethanol and centrifuged for 20 min at 4000 RPM. The presence of unreacted CD in the filtrate was measured spectroscopically using the extinction coefficient for CD (197,000 L mol^−1^ cm^−1^ at 834 nm). This procedure was repeated until the concentration of CD observed in the filtrate was less than 1 μM. The pellet was then washed again with 18 MΩ water and centrifuged to remove any remaining ethanol. The resulting CD-NP were removed from the filter using a spatula, dried, and stored at −20 °C (yield: 38.5 mg). For characterization, the NPs were resuspended in water at 10 mg/mL with sonication. 

### 2.3. Preparation of PAA NPs and Folic Acid Conjugate (PAA NP–FA)

First, 50 mg of lyophilized PAA nanoparticles were suspended in 3 mL of PBS (1×, pH 7.4) and sonicated for 10 min, resulting in a milky suspension. Then, 11 mg (57 μmol) of EDCI and 32 mg of Sulfo-NHS (147 μmol) were dissolved in 1.9 mL of MES buffer (100 mM, pH 6.0). To this solution, 2 mg (4.5 μmol) of folic acid, dissolved in 100 μL of DMSO, was added and stirred at room temperature for 20 min. Then, 100 μL of 1 N NaOH was added to the PAA nanoparticle solution to bring the final pH of the solution to 7 and stirred for 2 h at room temperature, protected from light. The excess folic acid, DMSO, and isourea by-product were removed by dialysis using cellulose dialysis tubing (12,000 kDa) in aqueous NaHCO_3_ (0.1 wt.%, pH 8) for 2 days and 18 MΩ water for 2 days. The solution of PAA NP–FA was removed from the tubing, lyophilized, stored at −20 °C (yield: 45 mg), and characterized.

### 2.4. Preparation of PAA NPs Conjugated with Cyanine Dye and Folic Acid (PAA NP–CD–FA)

The lyophilized PAA nanoparticles (50 mg) were suspended in 3 mL of PBS (1×, pH 7.4) and sonicated for 10 min, resulting in a milky suspension. Then, 11 mg (57 μmol) of EDCI and 32 mg of Sulfo-NHS (147 μmol) were dissolved in 1.9 mL of MES buffer (100 mM, pH 6.0). To this solution, 2 mg (4.5 μmol) of folic acid and 4 mg (4.1 μmol) of CD, dissolved in 100 μL of DMSO, were added and stirred at room temperature for 20 min. NaOH (100 μL of 1 N) was then added to the PAA nanoparticle solution to bring the final pH of the solution to 7 and was stirred overnight at room temperature, protected from light. The resulting mixture was transferred to an Amicon Ultra-4 100 kDa centrifuge filter and was centrifuged for 40 min at 4000 RPM to remove any DMSO, isourea by-product, unreacted cyanine dye, or folic acid. The remaining pellet was dispersed in 500 μL of 18 MΩ water using repeated pipetting and a spatula, diluted with 200-proof ethanol and centrifuged for 20 min at 4000 RPM. The supernatant was measured spectroscopically using the extinction coefficients for folic acid (25,100 L mol^−1^ cm^−1^ at 275 nm) and CD (197,000 L mol^−1^ cm^−1^ at 834 nm). This procedure was repeated until the concentrations of folic acid and CD observed in the filtrates were less than 1 μM. At this point, the pellet was washed a final time with 18 MΩ water and centrifuged to remove any remaining ethanol. The resulting FA–NP–CD was removed from the filter using a spatula, dried, and stored at 4 °C (yield: 37.2 mg). For characterization, the NPs were re-suspended in water at a concentration of 10 mg/mL with sonication. No significant change in the fluorescence intensity of CD with and without NPs was observed.

### 2.5. Preparation of Folic Acid–Hexamethyleneamine Amide

Folic acid (1.5 g; 3.14 mmol), dicyclohexylcarbodiimide (DCCI) (0.65 g; 3.14 mmol), 4-dimethylaminopyridine (DMAP) (0.38 g; 3.14 mmol), and 1,6-hexamethylene diamine (0.44 g; 3.77 mmol) were dissolved in the solvent mixture of 25 mL DMSO/2 mL dichloromethane and stirred for 2 days at room temperature. The solvent was removed under high vacuum. The residue was washed with dichloromethane to remove unreacted 1,6-hexamethylene diamine. The residue was washed with hexane (4 × 50 mL), and the fluffy powder thus obtained was used as such in the next step of the synthesis.

### 2.6. Preparation of a Carboxylic Acid-Functionalized Cyanine Dye

IR-820 (Sigma Aldrich, Waltham, MA, USA, CAS 172616-80-7) (252 mg; 0.2967 mmol) and *p*-mercapto-benzoic acid (283 mg; 1.8381 mmol) were reacted in 15 mL of dimethylformamide by following our own methodology [7], and the title compound was obtained in good yield (99.7 mg).

### 2.7. Preparation of a CD–FA Conjugate

Compound **2** (123 mg; 0.22 mmol), CD (150 mg; 0.15 mmol), and benzotriazol-1-yloxy)tris(dimethylamino)phosphonium hexafluorophosphate (BOP) (80 mg; 0.18 mmol) were dissolved in 40 mL of dimethylformamide (DMF), and triethylamine (Et_3_N; 12 drops) was then added. After the reaction mixture was stirred for 3 h, 0.5 mL of acetic acid was added. The solvents were removed under high vacuum and the residue was washed with n-hexane. The residue thus obtained was purified by HPLC using a Waters Symmetry Column, and the following gradient mobile phase: Solvent A: Acetonitrile (90%), DMSO (10%), and TFA (0.1%); solvent B: Water (90%), DMSO (10%), and TFA (0.1%); solvent C: Acetonitrile with TFA (0.1%). The initial mobile phase composition was 30% solvent A and 70% solvent B, which then graded to 100% solvent C at 30 min. The wavelength was set at 290 min and 799 nm (to detect the free folic acid and the folic acid–CD conjugate). The unreacted folic acid was eluted at 3–6 min. The major band eluted from 18–22 min was collected (Supporting Materials Figure S3).

### 2.8. Dynamic Light Scattering

The hydrodynamic diameter and size distribution measurements of the modified PAA nanoparticles were determined by dynamic light scattering (DLS) on a Nicomp 370 Submicron Particle Sizer (Nicomp, Santa Barbara, CA, USA). The NP solution was passed first through a 0.45 micron filter and then through a 0.2 micron filter immediately before measurement, placed in a borosilicate glass capillary tube and diluted with 1% Tween-80 in water to an intensity reading of 300 kHz (approximately 10 mg/mL). The readings were performed in a run set for 5 min. The volume-weighted Nicomp distribution proprietary analysis mode was used to determine the mean hydrodynamic diameter. 

### 2.9. Scanning Electron Microscopy

The morphology and size of the unformulated NPs were characterized by scanning electron microscopy (SEM). Lyophilized NP powder was placed on a mount with double-sided tape and crushed slightly to disrupt the macrostructure and to expose the fractured edges, following which it was coated with evaporated carbon. Images were taken on a Hitachi SU-70 SEM set at 5.0 kV and 3.5 mm × 100,000 magnification. TEM of blank and modified PAA NPs were not carried out due to the polymeric nature of these PAA-based NPs. As such, it was extremely difficult to create a thin layer/film of these NPs on TEM grids.

### 2.10. Zeta Potential

The zeta potential was determined using a dynamic light scattering instrument and Zeta Potential software (Brookhaven Instruments, Holtsville, NY, USA). NPs were suspended in 1% Tween-80 in water at 0.2 mg/mL, passed through a 0.2 micron filter, and measured in 10 replicates.

### 2.11. Spectrophotometry Analysis

To confirm the conjugation of folic acid and cyanine dye moieties in the PAA NPs, the UV-Vis spectrum of blank NPs (1 mg/mL) in 18 MΩ water filtered using a 0.8 micron filter was first collected from a range of 1000–200 nm (baseline standard). The absorption spectra of the known amounts of folic acid and the cyanine dye post-loading in PAA NPs were measured and the optical density (OD) values for both the compounds were determined (using the extinction coefficients of folic acid and cyanine dye analogue (25,100 L mol^−1^ cm^−1^ at 275 nm and 197,000 L mol^−1^ cm^−1^ at 834 nm), the concentrations of unreacted cyanine dye and folic acid were calculated and, subsequently, the amounts of free cyanine dye and folic acid post-loading in NPs were determined on the basis of their known amounts originally used for post-loading in NPs). Finally, the absorption spectrum of the FA–NP–CD conjugate in water (18 MΩ) was measured, and the concentrations of CD and FA were confirmed based on the OD values measured under similar conditions for the free CD and folic acid moiety, respectively.

### 2.12. In Vitro Folate Receptor Target-Specificity of CD–NP–FA

HT-1080 and KB cells (ATCC) were plated in triplicate at 1 million cells/well in 6-well tissue culture plates in 2 mL of RPMI-1640 cell culture media (Corning, Corning, NZ, USA) supplemented with 10% fetal bovine serum (Corning), 2 mM of l-glutamine (Corning), and 10,000 I.U./mL of penicillin, 10,000 g/mL of streptomycin (Corning), and was then allowed to grow overnight at 37 °C, 5% CO_2_, 100% humidity. The media was removed and 2 mL of folic acid-free RPMI-1640 (Gibco, Waltham, MA, USA) supplemented with 10% dialyzed FBS (Gibco), 2 mM of l-glutamine, 10,000 I.U./mL of penicillin, and 10,000 g/mL of streptomycin were added. Cells were incubated for 2 h. Folic acid was dissolved in 1 N NaOH to 100 mM (2000× stock) and 0.5 µL of this was added to each of the FA-designated wells to a final concentration of 50 µM (100× excess). Amino-functionalized PAA (AFPAA) conjugates and CD–FA were prepared in media and added at 50 µM (100× excess) to their respective wells, and 0.5 µL of NaOH was added to each well to control the pH effects. Cells were incubated for 2 min. FolateRSense680 (Perkin Elmer, Waltham, MA, USA) was prepared in PBS at 20 µM, and 25 µL/mL was added for a final concentration of 0.5 µM. Cells were incubated for 1 h, trypsinized, washed in PBS, and collected. FR680 uptake was measured on an Image Stream Flow Cytometer using IDEAS software v6.0.

### 2.13. In Vivo Folate Receptor Target-Specificity of CD–NP–FA

Female Nu/Nu mice (athymic nude mice, Charles River, Hopkinton, MA, USA) were fed a folic acid-free diet (Harlan) for 2 weeks to deplete FA stores. HT-1080 and KB cells were collected and re-suspended to 20 million cells/mL in serum-free RPMI-1640. One million cells in 50 µL were injected subcutaneously near the shoulder and grown for 1 week as the FA-free diet was continued. Folic acid was made up in 1 N NaOH to 100 mM, and then diluted to 1 mM in dextrose 5% in water (Baxter Healthcare Corporation, Deerfield, IL, USA) and injected as 200 µL/25 g mouse. Three mice per group were imaged on an IVIS Spectrum in vivo imaging system (Caliper Life Sciences, Hopkinton, MA, USA) with Living Images v4.3.1 proprietary software using 675 nm excitation and 720 emission filter sets. Regions of interest (ROIs) were drawn on the tumors, and fluorescence was calculated as total radiant efficiency [p/s]/[µW/cm^2^] or average radiant efficiency [p/s/cm^2^/sr]/[µW/cm^2^].

The in vivo experiments discussed in this manuscript were performed in compliance with all state, local, and federal laws and the PHS policy on the Human Care and Use of Laboratory Animals. This study was conducted in an AAALAC-accredited facility. The animal study was approved by the corresponding ethics committee of the institute.

## 3. Results and Discussion

To visualize the accumulation of PAA NPs in tumors, a CD with a carboxylic acid functionality, which has no tumor specificity by itself, was previously conjugated to the periphery of AFPAA NPs. Fluorescence images of Colon26 tumor-bearing BALB/c mice injected with the preparations showed that, in contrast to free CD, PAA NP–CD accumulated significantly in the tumor by 24 h. This result indicates that PAA NPs by themselves accumulate in tumors, probably through the enhanced permeability and retention (EPR) effect generated by leaky tumor vasculature and the lack of lymphatic drainage. The prepared PAA nanoparticles were characterized by absorbance spectroscopy; the size was determined using scanning electron microscopy (SEM) and dynamic light scattering (DLS). The hydrodynamic diameter and size distribution were determined using the Nicomp proprietary analysis mode.

**Figure 1:**
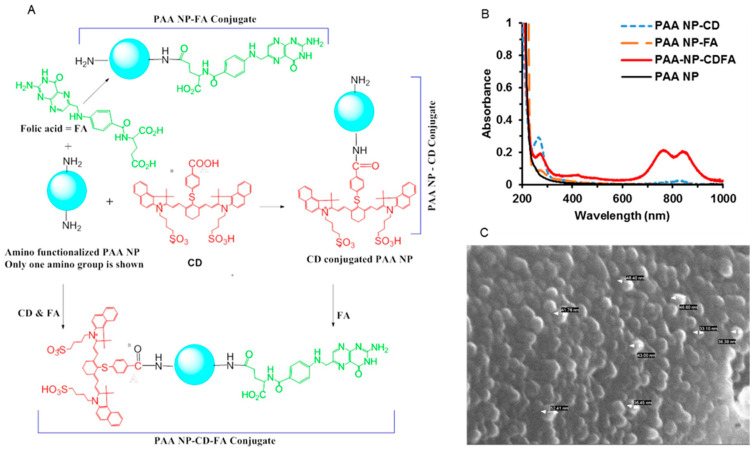
(**A**) Synthetic strategies for the preparation of folate-targeted nanoparticle (NPs) with and without cyanine dye (CD) moieties. (**B**) The electronic absorbance spectra of the nanoparticle constructs NP–CD, NP–folic acid (FA), and FA–NP–CD and blank NPs in 18 MΩ water (1 mg/mL). The NP–FA concentration is <1 mg/mL, due to poor solubility in 18 MΩ water. (**C**) SEM micrograph of FA–NP–CD at 150,000 × magnification. PAA, polyacrylamide.

**Figure 2:**
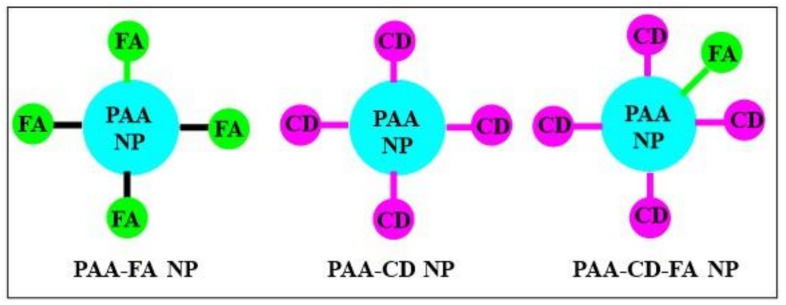
NPs conjugated either with folic acid (FA) or the cyanine dye (CD) or with both FA and CD.

In our attempt to develop a nano-construct with improved tumor-specificity, CD-conjugated folic acid-targeted NPs (FA–CD–NP) were developed in a stepwise fashion. Folic acid is a non-immunogenic vitamin necessary for DNA synthesis and repair. It is stable over a range of temperatures and pH levels and retains its ability to bind folate receptor after conjugation with a variety of drug candidates. Folic acid receptor [37] is known to be highly expressed on the cell surface of many tumor types, including breast, ovarian, and lung cancers. Therefore, to investigate the utility of PAA NPs in drug delivery, the dual-surface-conjugated PAA NP–CD–FA was prepared by using two different synthetic strategies (Figure 1 and Figure 2). In our stepwise approach, PAA NPs was first linked with the cyanine dye (CD) containing a carboxylic acid functionality, and the intermediate conjugate was then reacted with folic acid. In the second approach (a one-pot method), cyanine dye and folic acid were reacted with PAA NPs simultaneously, and the conjugate was isolated after purification. The number of the targeted moieties (FA) and the CD in the conjugate can be varied by varying the amount of each component introduced into the reaction mixture. However, stepwise controlled synthesis was found to be more reliable.

To confirm that both the cyanine dye and folic acid moieties were conjugated on surface of the NPs, the modified NPs were washed with ethanol until the filtrate was free from both the cyanine dye and the folic acid moieties, as measured by spectrophotometric analysis. This approach ensured that neither the cyanine dye nor the folic acid was post-loaded into the nanoparticles. The presence of cyanine dye and folic on the surface-modified PAA nanoparticles was determined spectrophotometrically. The characteristic absorption maximum of folic acid was observed at 290 nm in the NP–FA and FA–NP–CD nano-constructs. Similarly, the presence of cyanine dye covalently linked with amide bonds at the surface of the nanoparticle was also confirmed by UV-Vis analysis, exhibiting absorption bands for folic acid and CD (Figure 1B).

The size and morphology of the lyophilized NP constructs NP–FA, NP–CD, and FA–NP–CD were characterized using SEM (Figure 1C). The nanoparticles were mounted and crushed to disturb the smooth macrostructure and to expose the sheared edges and contours of the nanoparticles. The lyophilized nanoparticles appeared circular and uniform in shape (Supporting Materials Figure S1). The hydrodynamic diameter and size distribution of the modified NPs formulated in 1% Tween-80 were characterized by DLS and appeared to be consistent in size, similar to PAA-based NPs previously reported.

The size of the modified nanoparticles ranged from 52.9 to 59.2 nm for NP–CD, NP–FA, and FA–NP–CD. The zeta potential observed for the NPs is consistent with similar preparations and can be attributed to the primary amines present (Table 1; also see Supporting Materials Figure S2). After conjugation of cyanine dye and folic acid to the PAA NPs, the observed zeta potential of NP–FA, NP–CD, and FA–NP–CD were near neutral. This is most likely due to the chemical modification of the amine groups (Table 1).

The results shown in Table 1 are the hydrodynamic diameter and size distribution of the modified NPs dispersed in 1% Tween-80 water (solution phase), determined by dynamic light scattering (DLS) on a Nicomp 370 Submicron Particle Sizer (Nicomp, Santa Barbara, CA, USA), and appeared to be consistent in size to PAA-based NPs previously reported. The NP solution was syringe-filtered first through a 0.45 micron filter and then through a 0.2 micron filter immediately before measurement, placed in a borosilicate glass capillary tube and diluted with 1% Tween-80 in water to an intensity reading of 300 kHz (approximately 10 mg/mL). The volume-weighted Nicomp distribution proprietary analysis mode was used to determine the mean hydrodynamic diameter of the polydispersed samples.

DLS measures the hydrodynamic diameter, and compared to electron microscopy, the mean diameter in DLS is higher as it takes into account the surface modification. The hydrodynamic size provides information about the solvent layer that assembles around the nanoparticles. A larger apparent diameter indicates a greater ability to arrange a corona of solvent molecules around the nanoparticle, and such nanoparticles are likely to have a higher zeta potential and are more likely to remain dispersed over time (Supporting Materials Figure S2).

### 3.1. Folate Receptor Target-Specificity of Nanoparticles

The folic acid receptor (FR) is a target for the delivery of cancer therapeutics because of its elevated expression in many cancer cells [37]. FA was tested as a potential ligand for enhanced tumor-delivery NPs. To demonstrate the specificity of FA binding in vitro and in vivo, the NIR fluorescent FR ligand FolateRSense^TM^680 (FR680, Perkin-Elmer, Waltham, MA, USA) was used to confirm the folate receptor-binding ability. In vitro binding of FR680 was assessed by flow cytometry using KB cells (high expression of FR) and HT-1080 cells (low expression of FR). 

**Figure 3:**
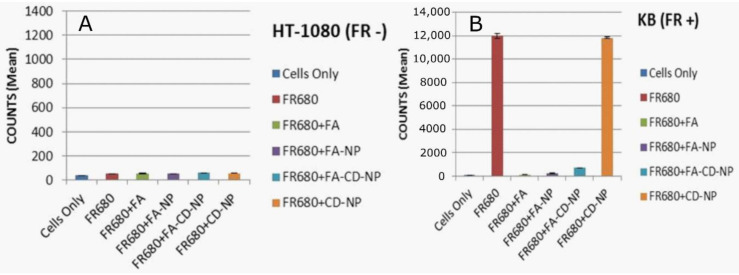
In vitro accumulation and competition assays for binding to folate receptors (FRs) indicate FA–CD–NP specificity for the receptor. Flow cytometry confirms a high degree of labeling of folate receptor-positive KB cells (right: mean count 11,987, (**B**) over folate receptor-negative HT-1080 cells (left: mean count 54, (**A**). Folic acid, FA–NP, and FA–NP–CD showed effective competition for binding in KB cells. As expected, NP–CD did not inhibit FR680 binding in the folate receptor-positive cell line.

The relative target-specificity of FA–NP and FA–NP–CD was investigated in tumor cell lines with variable levels of FR expression, showing that NPs with surface-conjugated FA have specificity toward folate receptors, which was further confirmed by inhibition assay by saturating the folate receptors with FR680 first before adding either FA–NP or FA–NP–CD. This reduced the uptake of the dye significantly, and, as expected, NPs without the presence of FA did not reduce the fluorescence intensity of the dye in folate receptor-positive cells (Figure 3).

**Figure 4:**
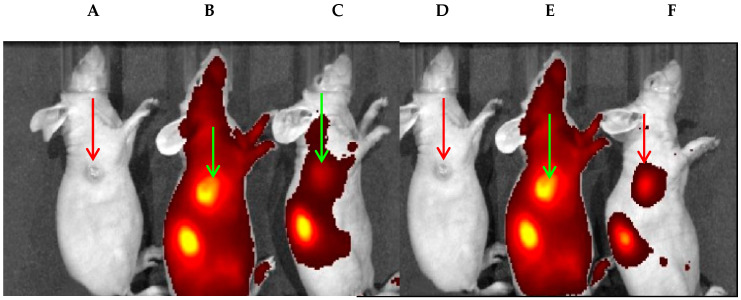
(**A**,**D**) Female Nu/Nu mice bearing KB+ tumor size: ~65 mm^3^. (**B**) Mice injected with FR680 and imaged at 24 h post-injection. (**C**) A mouse was injected with FA (56-fold higher dose) first before injecting FR680 (0.08 nmoles/g). (**E**) A mouse was injected with NP–CD–FA and imaged after 24 h post-injection. (**F**) The FA in ~56-fold excess was injected first, before administrating PAA NP–FA, and imaged at 24 h post-injection. The reduced tumor fluorescence intensity in the mice in (**C**) and (**F**) suggests the target specificity of FR680 and FA–NP–CD (PAA-based NPs).

The in vivo target-specificity of FA–NP–CD was also confirmed by inhibition assay in mice implanted with KB+ tumors (size: 4–5 mm). The near-infrared optical imaging was performed at 2, 4, and 24 h post-injection (only 24 h whole-body images are shown in Figure 4). The multispectral imaging system IVIS Spectrum (Perkin-Elmer), along with Living Image v4.3.1 (image acquisition and analysis software), were used. A region of interest (ROI) was defined to measure FR680 uptake.

### 3.2. CD–FA Conjugate Shows Limited Target-Specificity

**Figure 5:**
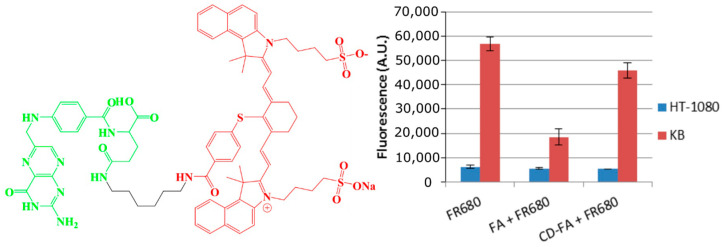
The CD–FA conjugate (**left**) used in an in vitro competition assay was unable to block the uptake of FR680 (**right**) or folic acid in KB cells. FR680 uptake in FR^low^ HT-1080 cells was not affected in the presence of free FA or the CD–FA conjugate.

To demonstrate the advantages of PAA NPs in developing FA-targeted fluorophores, the CD used for conjugation with NPs was also conjugated with FA containing amino functionality by following a similar synthetic approach discussed for the FA–NP conjugate. The CD–FA thus obtained was also tested for target specificity following the cell uptake inhibition approach in the presence of free FA. The results summarized in Figure 5 indicate that compared to FR680, the CD–FA conjugate showed limited folate receptor target-specificity and shows the advantages of NPs in achieving target-specificity of the FA–NP–CD construct.

## 4. Conclusions

In summary, this report illustrates the unique features of PAA nanoparticles that make them suitable for receptor-targeted imaging: (a) Their non-toxic, biocompatible, and biodegradable nature; (b) their synthetic reproducibility; (c) their prolonged presence in blood circulation; (d) their ability to introduce the desired functional groups for the conjugation of targeting agents; (e) their large surface area for loading a large number or multiple types of tumor-targeting ligands. However, detailed toxicity in larger animals at various doses, following the FDA guidelines, remain to be addressed before evaluating PAA-based nanoparticles for cancer imaging/therapy in Phase I human clinical trials. Studies are underway to post-load long-wavelength absorbing photosensitizer(s) and chemotherapeutic agent(s) at the desired ratio(s) in targeted nanoparticles for NIR fluorescence-guided combination therapy of folate receptor-positive tumors. The versatile nature of PAA-based NPs provides an opportunity to introduce desired targeting group(s) at the surface of the nanoparticles for imaging and therapy of various cancer indications.

## Figures and Tables

**Table 1:** Hydrodynamic diameters and zeta potential of PAA-based nanoparticles ^a^.

	NPs (PAA)	NP–FA	NP–CD	FA–NP–CD
Diameter (nm) ^b^	63.1 ± 7.2	58.4 ± 6.6	52.9 ± 3.3	59.2 ± 6.3
Zeta potential (mV)	13.1 ± 0.5	0.0 ± 0.0	0.2 ± 0.5	0.0 ± 0.0

^a^ Measurements made in water with 1% Tween-80. ^b^ The volume-weighted Nicomp distribution proprietary analysis mode was used to determine the mean hydrodynamic diameter.

## Supplementary Materials

The following are available online at https://www.mdpi.com/2218-273X/10/12/1651/s1, Figure S1. Scanning electron microscopy (SEM) images of (A) polyacrylamide (PAA) nanoparticle (NP)–cyanine dye (CD) and (B) PAA NP–folic acid (FA) at 20,000× magnification. Sizes range from 75 to 125 nm and 25 to 65 nm, respectively; Figure S2. The zeta potential for (A) blank PAA NPs, (B) PAA NP–CD, (C) PAA NP–FA, and (D) PAA NP–FA–CD in 1% Tween-80 in water. The zeta potentials observed were 13.1, 0.2, 0.0, and 0.0 mv for (A), (B), (C), and (D), respectively; Figure S3. Preparative reverse-phase HPLC run of the CD–FA synthesis reaction on a C18 symmetry column, using mobile phase ACN 30% (90% ACN, 10% DMSO, and 0.1% TFA): H_2_O 70% (90% H_2_O, 10% DMSO, and 0.1% TFA) run on a 30 min gradient to ACN 100% (90% ACN, 10% DMSO, and 0.1% TFA). The 799 nm channel indicates the presence of cyanine dye, both free form and conjugated CD–FA. The integration shows unreacted cyanine dye at 3–6 min, the CD–FA conjugate at 18–22 min, and MEOH wash (10 min gradient to 90% MEOH, 10% DMSO, and 0.1% TFA) starting at 40 min. The major fraction (18–22 min) was collected and used for in vitro/in vivo studies.
