# Supplementary material for: Targeted Nanoparticles for Fluorescence Imaging of Folate Receptor Positive Tumors
Source: Biomolecules. 2020 Dec 9;10(12):1651. doi: 10.3390/biom10121651 (PMC7764199; doi:10.3390/biom10121651)
Supplement: Supplementary file 1 [file biomolecules-10-01651-s001.pdf]

Supporting Material

Targeted Nanoparticles for Fluorescence Imaging of Folate Receptor Positive Tumors

Aimee J Marko[a], Ballabh M. Borah[b], Kevin E Siters[b], Penny Joshi[a], Joseph R. Missert[a], Joseph Cacaccio[a], Meden F. Isaac-Lam[c] and Ravindra K. Pandey[a]

[a] Photodynamic Therapy Center, Cell Stress Biology, Roswell Park Comprehensive Cancer Center, Buffalo, NY 14263

[b] Photolitec, LLC, 73 High Street, Buffalo, NY 14203

[c] Department of Chemistry and Physics, Purdue University, Northwest, Westville, IN 46391

Contents:

1. **Figure S1:** SEM images of PAA NP-CD and PAA NP-FA.
2. **Figure S2:** Zeta potential of PAA nanoparticle constructs.
3. **Figure S3:** Preparative Reverse-Phase HPLC profile of the CD-FA conjugate reaction mixture.

A)

B)

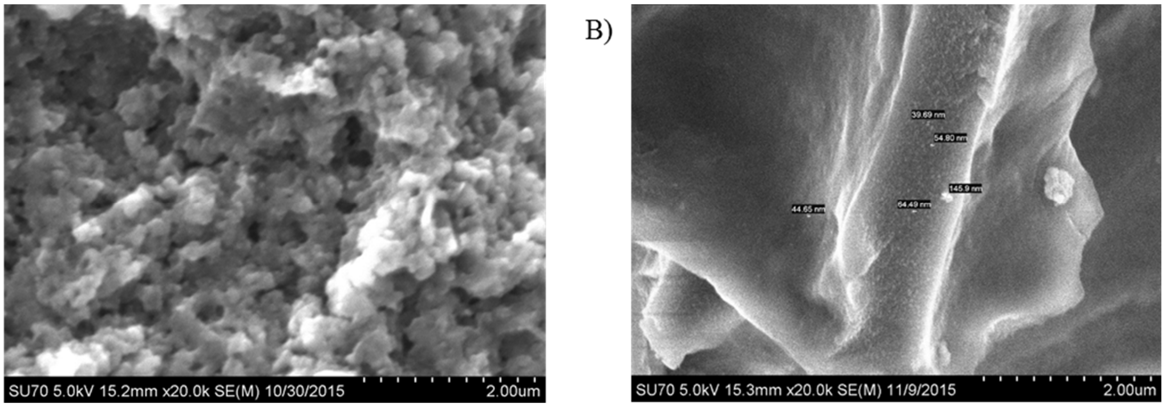

Figure S1: SEM images of A) PAA NP-CD and B) PAA NP-FA at 20,000x magnification. Sizes range from 75-125 nm and 25-65 nm, respectively.

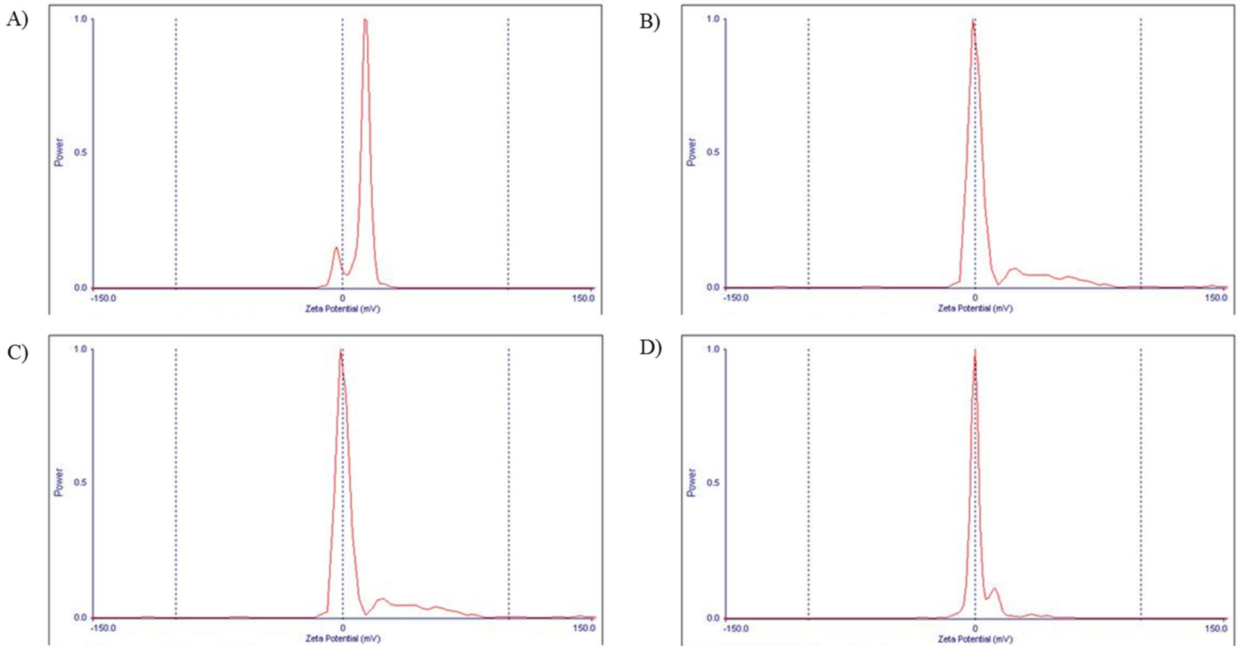

Figure S2: The zeta potential for A) Blank PAA NPs, B) PAA NP-CD, C) PAA NP-FA and D) PAA NP-FACD in 1% Tween 80 in water. The zeta potential observed are 13.1 mv, 0.2 mv, 0.0 mv and 0.0 mv for A, B, C and D, respectively

SampleName 2015-05-22 AIMEE 2

Date Acquired 5/22/2015 11:48:00 AM EDT

Column_info

Processing Method test

Injection Volume 200.00 uL

Channel Description 2998 (200-799)nm

Mobile_Phase

Channel Name 799 nm

Flow rate

Acq Method Set FA_CDms

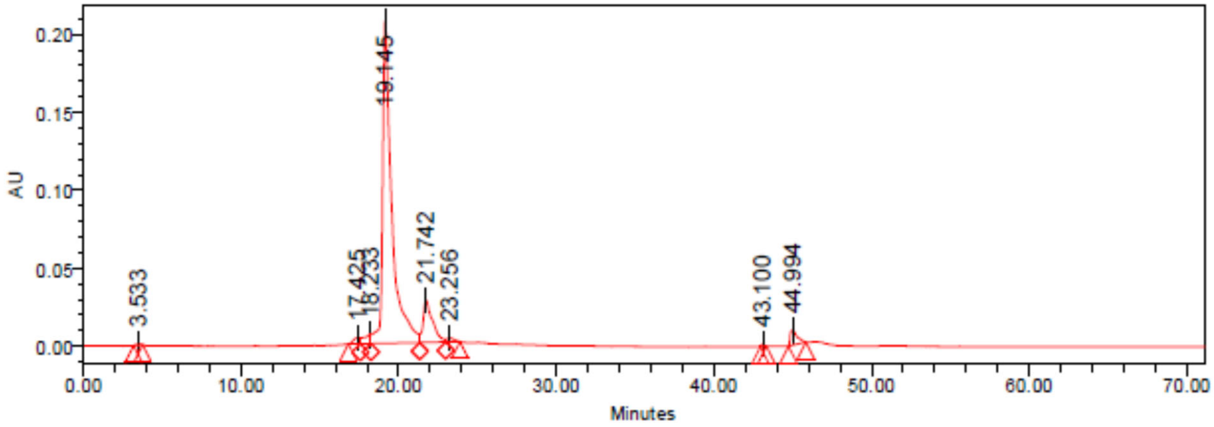

Peak Results

	Name	RT	Area	% Area	Height	% Height
1		3.533	15331	0.15	922	0.36
2		17.425	105363	1.04	3803	1.48
3		18.233	182950	1.80	5707	2.22
4		19.145	8334161	82.15	206227	80.09
5		21.742	1112903	10.97	27089	10.52
6		23.256	79822	0.79	2486	0.97
7		43.100	15314	0.15	1667	0.65
8		44.994	299037	2.95	9581	3.72

Figure S3. Preparative Reverse-Phase HPLC run of the CD-FA synthesis reaction on a C18 Symmetry column, using mobile phase ACN 30% (90% ACN, 10% DMSO, 0.1% TFA): H_2O 70% (90% H_2O, 10% DMSO, 0.1% TFA) run on a 30 min gradient to ACN 100% (90% ACN, 10% DMSO, 0.1% TFA). The 799 nm channel indicates presence of cyanine dye, both free form and conjugated CD-FA. The integration shows unreacted cyanine dye at 3-6 minutes, CD-FA conjugate at 18-22 minutes, and MEOH wash (10 min gradient to 90% MEOH, 10% DMSO, 0.1% TFA) starting at 40 minutes. The major fraction (18-22 min) was collected and used for in vitro/in vivo studies.
